# Resistance to Gray Leaf Spot of Maize: Genetic Architecture and Mechanisms Elucidated through Nested Association Mapping and Near-Isogenic Line Analysis

**DOI:** 10.1371/journal.pgen.1005045

**Published:** 2015-03-12

**Authors:** Jacqueline M. Benson, Jesse A. Poland, Brent M. Benson, Erik L. Stromberg, Rebecca J. Nelson

**Affiliations:** 1 School of Integrative Plant Sciences, Cornell University, Ithaca, New York, United States of America; 2 Department of Agronomy, Kansas State University, Manhattan, Kansas, United States of America; 3 203 Solutions LLC, Baltimore, Maryland, United States of America; 4 Department of Plant Pathology, Physiology and Weed Science, Virginia Polytechnic Institute, Blacksburg, Virginia, United States of America; Max Planck Institute for Terrestrial Microbiology, Germany

## Abstract

Gray leaf spot (GLS), caused by *Cercospora zeae-maydis* and *Cercospora zeina*, is one of the most important diseases of maize worldwide. The pathogen has a necrotrophic lifestyle and no major genes are known for GLS. Quantitative resistance, although poorly understood, is important for GLS management. We used genetic mapping to refine understanding of the genetic architecture of GLS resistance and to develop hypotheses regarding the mechanisms underlying quantitative disease resistance (QDR) loci. Nested association mapping (NAM) was used to identify 16 quantitative trait loci (QTL) for QDR to GLS, including seven novel QTL, each of which demonstrated allelic series with significant effects above and below the magnitude of the B73 reference allele. Alleles at three QTL, *qGLS1.04*, *qGLS2.09*, and *qGLS4.05*, conferred disease reductions of greater than 10%. Interactions between loci were detected for three pairs of loci, including an interaction between *iqGLS4.05* and *qGLS7.03*. Near-isogenic lines (NILs) were developed to confirm and fine-map three of the 16 QTL, and to develop hypotheses regarding mechanisms of resistance. *qGLS1.04* was fine-mapped from an interval of 27.0 Mb to two intervals of 6.5 Mb and 5.2 Mb, consistent with the hypothesis that multiple genes underlie highly significant QTL identified by NAM. *qGLS2.09*, which was also associated with maturity (days to anthesis) and with resistance to southern leaf blight, was narrowed to a 4-Mb interval. The distance between major leaf veins was strongly associated with resistance to GLS at *qGLS4.05*. NILs for *qGLS1.04* were treated with the *C*. *zeae-maydis* toxin cercosporin to test the role of host-specific toxin in QDR. Cercosporin exposure increased expression of a putative flavin-monooxygenase (FMO) gene, a candidate detoxification-related gene underlying *qGLS1.04*. This integrated approach to confirming QTL and characterizing the potential underlying mechanisms advances the understanding of QDR and will facilitate the development of resistant varieties.

## Author Summary

Gray leaf spot (GLS), a necrotrophic, foliar fungal disease of maize, contributes to maize yield losses worldwide. We identified and characterized regions of the maize genome that confer resistance to GLS and gained insight into the mechanisms associated with these quantitative trait loci (QTL). We provide evidence for structural and detoxification-related mechanisms underlying quantitative resistance. The distance between major veins of the maize leaf was positively correlated with the quantity of fungal conidiophores (reproductive structures) produced. Four of the GLS QTL were associated with inter-vein distance, and co-localization was confirmed for one of these QTL in near-isogenic lines. In addition, up-regulation of a putative detoxification-related flavin-monooxygenase gene was correlated with a fine-mapped QTL. Plant breeding decisions regarding development and deployment of disease resistance traits can be improved with better understanding of the mechanisms underlying quantitative disease resistance.

## Introduction

Plants resist attack by many plant pathogens by inducing localized cell death. This strategy can provide effective defense against pathogens that require living host tissue (biotrophs or hemibiotrophs) and is the basis for most monogenic resistance. However, it is not effective for pathogens that feed on dead tissue (necrotrophs) [1]. For this reason, complete, single-gene resistance is typically unavailable for diseases caused by necrotrophic pathogens [2,3]. Gray leaf spot (GLS), a foliar disease of maize caused by the polycyclic pathogens *Cercospora zeae-maydis* and *Cercospora zeina* [4,5], is a necrotrophic pathogen that is mostly controlled by quantitative forms of host plant resistance. Understanding the mechanisms underlying resistance to GLS may further elucidate the biology of host-pathogen interactions since there are contrasting mechanisms of defense against necrotrophic and biotrophic pathogens [6]. GLS is one of the most important maize diseases in the United States and in maize-growing regions worldwide [7]. It can cause yield losses over 70% due to associated severe blighting, stalk deterioration and lodging [8,9].

Although it is critical for the management of most plant diseases (not only those caused by necrotrophic pathogens), the genetic basis of quantitative disease resistance is not well understood. A better understanding of the genetic architecture and the underlying genes and mechanisms could contribute to crop improvement and disease management. Previous QTL studies have improved understanding of the genetic architecture by identifying regions of the genome that confer resistance to GLS [4,10–22]. The aim of this study was to improve understanding of the locations, sizes and interactions among loci controlling GLS resistance using nested association mapping (NAM) [23] and to develop hypotheses concerning the underlying mechanisms of resistance.

Quantitative resistance can be divided into a range of sub-phenotypes at the macroscopic and microscopic levels. These phenotypes include reduction in total number of infections, reduction in lesion expansion, reduction of sporulation, lengthening of the latent period, and increasing the number of propagules necessary to establish infection [24]. Individual QTL may differentially affect specific sub-phenotypes [25]. Poland et al. [26] provided six hypotheses regarding mechanisms that underlie QDR loci: (1) genes that underlie plant development and architecture, (2) genes with mutations or allelic changes in genes involved in basal defense, (3) genes involved in secondary metabolite production known to fend off pathogen attacks, (4) genes involved in signal transduction, (5) weak forms of *R*-genes, and (6) genes previously unassociated with pathogen defense. Cloning of several disease QTL has provided hints about the underlying molecular mechanisms involved in resistance [27–32].

Poland’s third hypothesis above indicates that such mechanisms might include those involved in chemical warfare between plant pathogens and their hosts. For example, genes for resistance might mitigate the damaging effects of microbial compounds on host tissues. Plant pathogens produce an array of toxic molecules and enzymes that aid in host infection [33]. These products come in diverse chemical forms and are either host non-specific or specific. *Cercospora zeae-maydis*, the predominant causal agent of GLS in the United States, produces the non-host-selective toxin cercosporin. Cercosporin is a photo-activated perylenequinone that converts molecular oxygen to active oxygen species, including singlet oxygen [34]. Plant carotenoids play a key role in quenching singlet oxygen before it damages the chloroplast or other machinery within the plant cell [35] and are hypothesized to play a role in defense against toxins that produce reactive oxygen species [36]. Additional enzymes and metabolites, such as oxidoreductases and secondary metabolites with antioxidant properties, may be involved in cercosporin detoxification or in reducing damage caused by cercosporin [37–39]. We explored the hypothesis that detoxification could play a role in GLS resistance by analyzing the annotation and expression of genes underlying one of the detected GLS loci.

It has also been proposed that some genes conferring quantitative disease resistance are related to morphological and developmental processes such as leaf structure and flowering time [40,41]. GLS symptoms include tan-colored, rectangular-shaped lesions that elongate parallel to the leaf venation. Lesions appear to be bounded by major veins [42]. We hypothesized that inter-vein distance (IVD) could act as a resistance-related parameter for GLS, because the width of GLS lesions is defined by IVD. Although there is evidence that lesion expansion can contribute significantly to disease epidemics caused by *Cercospora spp*., to our knowledge, no studies have examined the relationship between leaf venation structure and lesion development.

## Results

### Heritability of resistance

The NAM population was screened for GLS in Blacksburg, VA, and exhibited a wide range of disease levels among parental lines within and among populations (Table 1). Parental line AUDPC varied from 35.2 to 51.5 at the point of maximum variance identified among BLUPs. Mean NAM population AUDPC was 42.6 (*σ* ^*2*^ = 13.2). Broad sense heritability on an individual plot basis was 0.72, while on a line mean basis, correcting for the unbalanced design of the experiment, it was 0.83 [43]. Additive genetic variance was responsible for 52% of the phenotypic variance as determined by calculating the correlation between the sum of all parental effects and the parental phenotypic BLUPs.

**Table 1:** AUDPC summary statistics for the nested association mapping population.

Parents	Mean	SE	SD	V	Range	Min	Max	Count
B97	44.72	0.28	3.38	11.39	16.03	34.75	50.78	150
CML103	40.45	0.28	3.37	11.35	18.27	32.14	50.41	149
CML228	39.29	0.29	3.46	12.00	16.18	31.76	47.95	147
CML247	38.79	0.24	2.94	8.67	17.42	29.61	47.03	150
CML277	36.78	0.33	3.87	15.00	18.86	26.94	45.80	138
CML322	43.25	0.24	2.91	8.49	13.59	37.46	51.05	148
CML333	38.68	0.37	4.45	19.82	27.92	28.61	56.53	148
CML52	35.18	0.26	3.17	10.03	15.39	28.81	44.20	148
CML69	40.68	0.28	3.40	11.55	21.59	27.75	49.34	149
HP301	46.81	0.25	3.01	9.05	15.28	39.11	54.39	150
IL14H	51.49	0.34	4.15	17.19	24.50	36.75	61.25	150
KI11	45.91	0.26	3.20	10.25	16.31	38.45	54.76	150
KI3	39.75	0.38	4.27	18.22	24.22	26.84	51.06	125
KY21	44.34	0.31	3.82	14.58	20.58	34.27	54.85	147
M162W	42.80	0.23	2.83	8.03	13.04	36.27	49.31	150
M37W	43.87	0.32	3.85	14.82	27.47	33.20	60.67	149
MO17	46.29	0.24	2.91	8.47	15.01	37.99	52.99	150
MO18W	37.11	0.30	3.63	13.20	19.69	29.31	49.00	150
MS71	48.33	0.35	4.30	18.47	21.58	36.48	58.06	148
NC350	36.66	0.30	3.69	13.61	19.41	27.73	47.14	150
NC358	39.14	0.33	4.04	16.32	20.24	29.87	50.10	150
OH43	47.04	0.22	2.74	7.51	14.83	38.27	53.11	150
OH7B	48.41	0.35	4.25	18.04	21.20	36.82	58.02	150
P39	48.40	0.28	3.37	11.38	14.10	41.27	55.37	150
TX303	39.51	0.25	3.10	9.60	16.49	32.36	48.85	150
TZI8	43.70	0.44	5.06	25.57	24.41	30.33	54.73	132
All Parents	39.97	1.26	6.56	43.01	22.27	28.26	50.53	27

Descriptive statistics for area under the disease progress curve within the nested association mapping sub-populations (sub-population is indicated by the non-B73 parent) and among the parental lines.

SE = Standard Error

SD = Standard Deviation

V = Variance

Min = minimum

Max = maximum

### Disease resistance loci and interactions

**Fig 1:**
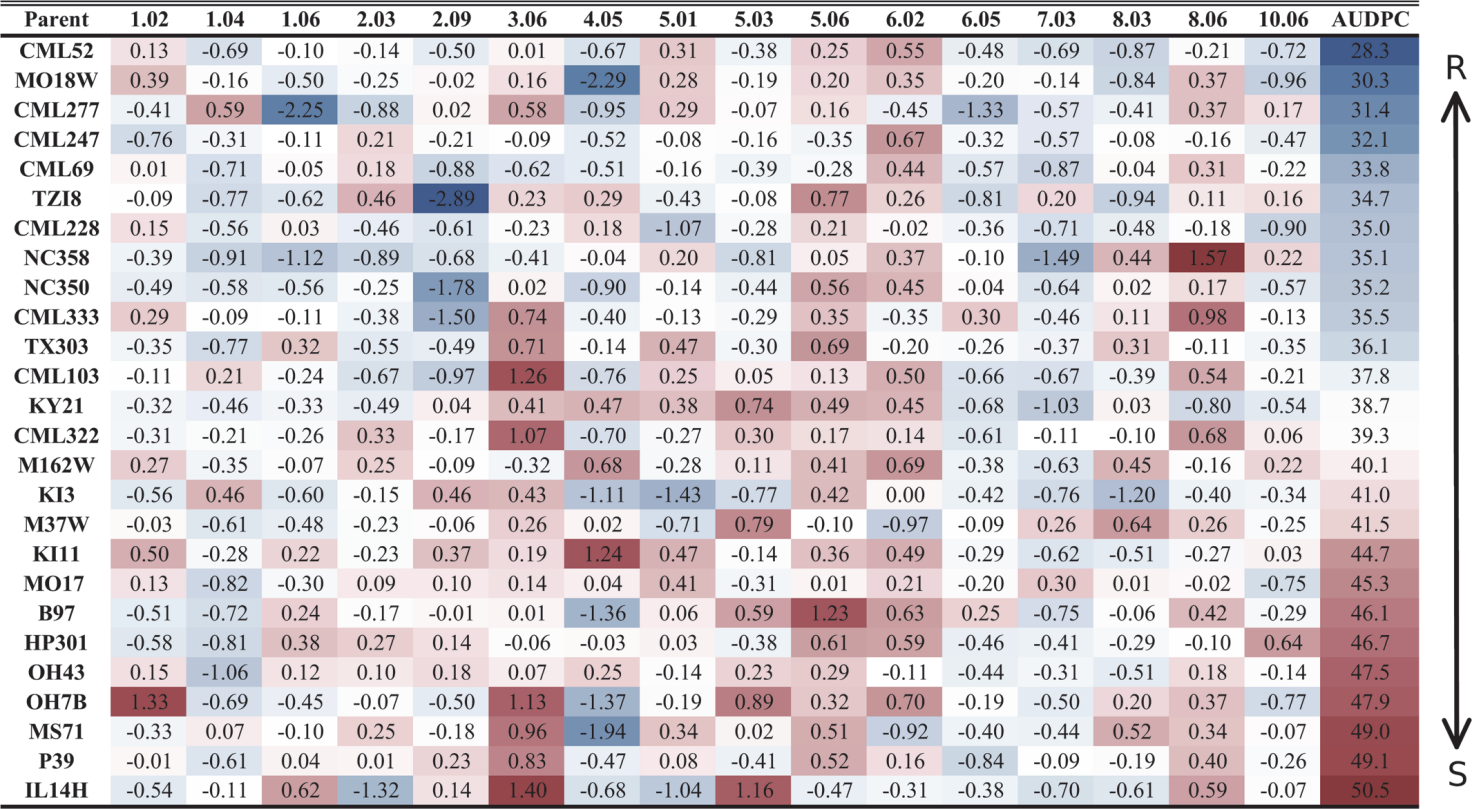
Parental allelic effects at quantitative trait loci. The bin number is listed in the respective column header between the first and last columns. Two sets of data are presented in this figure. Estimated allelic effects relative to the B73 allele of parental lines at *qGLSbin* for relative change in disease are listed. The values in the table are the coefficients of the general linear model parameter while the color coding is indicative of area under the disease progress curve. R = resistant; S = susceptible.

**Fig 2:**
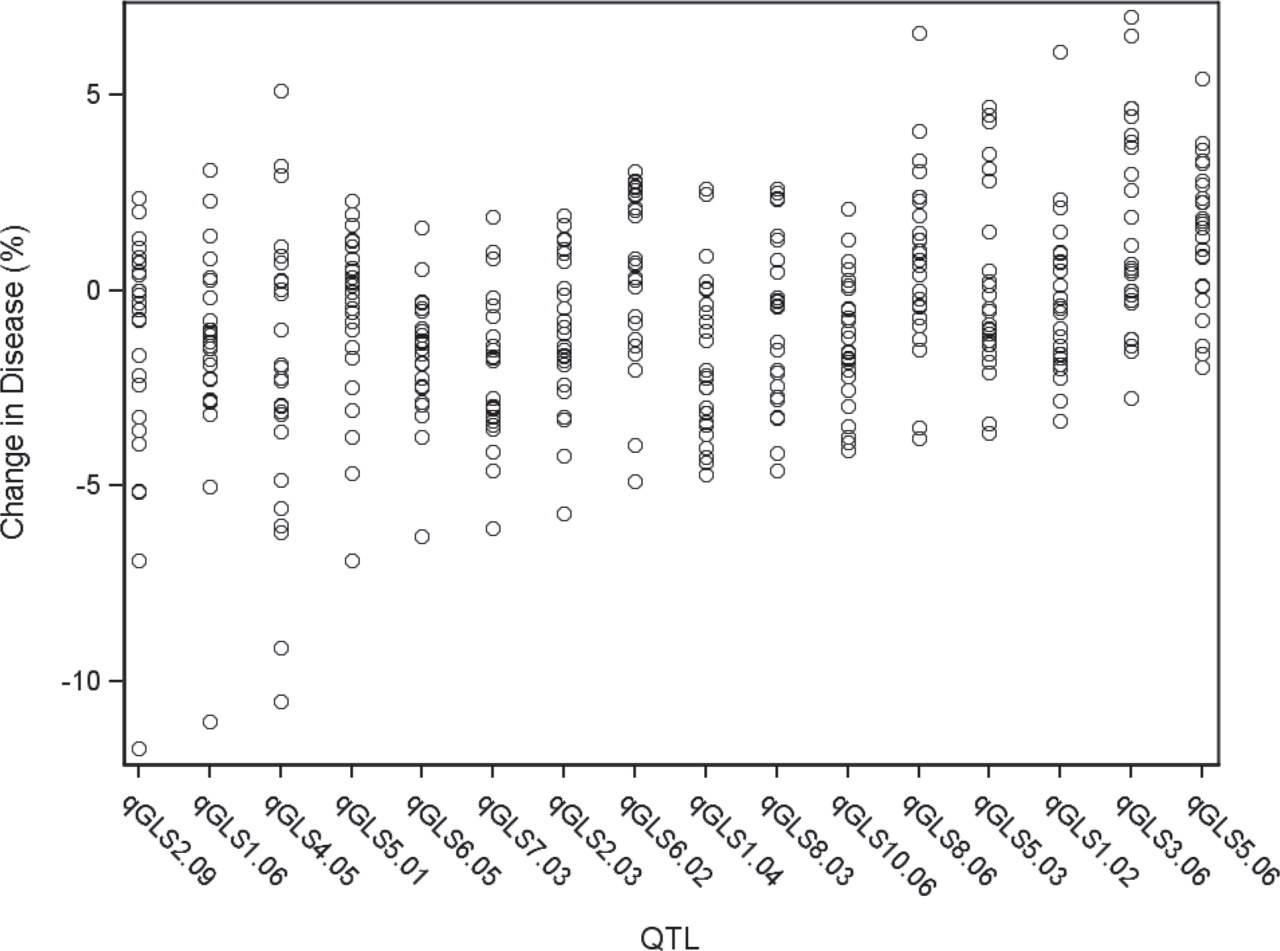
General linear model predicted percent change in disease across significant disease quantitative trait loci (QTL). Each circle indicates the predicted change in disease of a single allele. There are 26 circles for each QTL, each representating the allele from one of the nested association mapping parental sources.

A model selection approach to QTL mapping was used to identify loci that significantly described GLS disease progress. GLS QTL were designated as *qGLSbin#*_x_, where “q” indicates a QTL, bin# is replaced with the genomic bin location within which the selected markers were located, and “x” denotes the genotype source of the specified allele. Sixteen markers, located on nine of the 10 chromosomes, were selected by the model at *p*<4.3x10^–4^ (Table 2). The selection threshold was based on the q-value calculation for false discovery rate (FDR). Effect sizes across parental lines varied at each locus. The estimated effects on disease levels of each parental allele at each QTL relative to the B73 allele are listed in Fig. 1. Across the set of QTL, each parental line had susceptible, resistant and neutral allelic effects relative to B73. Significant model-selected QTL explained 78.5% of the phenotypic variation within the population, while inclusion of all QTL (*p*<0.05) explained 82.2% of the variation. The parental LSM of AUDPC for each locus was used to estimate the effect of each allele on disease levels. Alleles at three loci, *qGLS1.04*, *qGLS2.09* and *qGLS4.05*, were predicted to confer disease reduction of greater than 10% (Fig. 2). The greatest change in disease reduction was predicted to be 11.7% for the TZI8 allele at *qGLS2.09*.

**Table 2:** Summary of resistance to gray leaf spot (GLS) in the nested association mapping population.

GLS QTL Designation	Marker Name	p-value	Chr	Position (bp)	CI (cM)	Co-localizing GLS QTL	Other NAM QTL
qGLS1.02	PZB01957.1	2.94E-10	1	22,892,866–28,421,841	7.6	**	NLB
qGLS1.04	PHM5098.25	1.06E-20	1	56,747,253–83,780,725	12.1	**	DTA, NLB^+^, SLB
qGLS1.06	PHM1968.22	3.15E-11	1	161,027,952–208,733,347	28.9	[12,15,16,63]	NLB, SLB
qGLS2.03*	PHM6111.5	6.37E-07	2	14,836,855–22,999,224	14.3	[11,16]	DTA
qGLS2.09*	PZA02727.1	1.94E-34	2	212,537,417–235,852,920	35.2	[18,19]	DTA, SLB
qGLS3.06	PZA00186.4	8.31E-19	3	143,898,953–180,504,690	26.3	**	DTA, NLB^+^, SLB^-^
qGLS4.05	fea2.3	4.70E-44	4	9,759,854–178,889,832	64.8	[10,16]	DTA, NLB, SLB
qGLS5.01	PZA02753.1	1.27E-07	5	5,928,250–7,985,979	8.1	**	DTA
qGLS5.03*	PZA02792.26	1.88E-06	5	15,138,119–30,994,484	10.8	[11,12,15,19,63]	SLB
qGLS5.06*	PZA02667.1	2.70E-08	5	192,167,921–207,708,797	21.9	**	DTA, NLB^+^, SLB
qGLS6.02	PZA00214.1	3.98E-06	6	86,257,528–113,885,960	28.4	**	NLB
qGLS6.05	PZA02673.1	2.00E-08	6	118,087,791–147,224,252	15.8	**	NLB^+^, SLB
qGLS7.03	PZA00986.1	8.42E-15	7	13,174,365–142,783,202	35.4	[11,12,20]	DTA, NLB^+^, SLB
qGLS8.03*	PZA01470.1	2.08E-12	8	23,769,876–101,178,933	7.6	[19]	DTA, SLB
qGLS8.06*	PZA03651.1	2.96E-06	8	135,091,499–156,907,035	12.6	[16,63]	NLB, SLB
qGLS10.06*	PZA02663.1	2.62E-05	10	136,941,040–142,193,827	12.4	[11,20]	DTA, NLB^+^, SLB

Quantitative trait loci (QTL) were identified by nested association mapping of GLS resistance. Model selection results are given in relation to the co-localizing QTL associated with previously published studies. *p*<0.0001, Chr = Chromosome

CI = Confidence Interval

DTA = Blacksburg (Whitethorne Farms)-specific days to anthesis

Southern Leaf Blight (SLB) QTL Source: [46]

Northern Leaf Blight (NLB) QTL Source: [40]

Asterisks indicate better resolved QTL

double asterisks denote QTL reported here for the first time

^+,-^ indicates significant positive, negative association of allelic effects, respectively.

All possible two-way interactions were entered one-by-one as predictors of AUDPC in the GLM model containing main-effect QTL and the parameters DTA and population. Three allelic interactions were detected. One pair of interacting loci involved two markers within significant disease QTL confidence intervals. There was a significant interaction between the two QTL significant for disease at *qGLS4.05* and *qGLS7.03*. The other two pairs involved interactions between significant and non-significant markers at *p*<1.0x10^–5^. One of these interactions was between *qGLS2.03* and maize bin 7.06 (175.8 Mb) while the other was between *qGLS6.02* and *8.02* (12.3 Mb).

In addition to joint linkage, The NAM population was used to identify loci associated with resistance to GLS based on a genome-wide association study (GWAS). GWAS revealed a total of 145 single-nucleotide polymorphisms (SNPs) that were significantly associated with the GLS phenotype (S1 Table). One or more genes were identified within a 20 kb window of the significant association (hit) for 63 of the 145 GWAS hits (S1 Table). Characterized functional annotations based on the gene sequence provided in the maize B73 reference genome (V2) were detected for 41 of the 63 genes located within 20 kb of a GWAS hit. A proportions test was conducted for the gene ontology (GO) terms in each of these 41 genes to determine whether the observed frequency of the evaluated gene type associated with GLS QTL exceeded the frequency that would be expected at random, based on the frequency of the evaluated gene type in the maize genome (Table 3). Genes related to cellular energy, development, and secondary metabolites were statistically overrepresented categories with *p*-values of 2.22 x 10^–10^, 2.24 x 10^–15^, and 1.23x10^–3^, respectively.

**Table 3:** Summary statistics of functionally annotated and categorized genome wide association hits.

Category	Genome (count)	GLS GWAS (count)	SE	z-value	p-value
Cell Cycle	25777	13	0.077	1.155	8.76E-01
Cell Signal	21016	2	0.073	3.837	1.00E+00
Cellular Energy	805	5	0.018	-6.237	2.22E-10
Development	205	3	0.009	-7.841	2.24E-15
Membrane Transporter	2896	0	0.033	1.399	9.19E-01
Pathogen Defense Related	4590	4	0.040	-0.626	2.66E-01
Primary Metabolite	204	1	0.009	-2.391	8.39E-03
Secondary Metabolite	4604	8	0.041	-3.027	1.23E-03
Structural Components	3442	5	0.035	-1.916	2.77E-02

Significant GWAS hits were functionally annotated using basic local alignment search tool. These annotations were categorized into biological groups, which include: structural components, secondary metabolites, primary metabolites, genes previously implicated in pathogen-defense, membrane transporter, development, cellular energy, cell signaling, and cell cycle. The genes on the inter-pro hit list (www.maizesequence.org) were also categorized into these same categories. The genome count indicates the total number of genes within the genome in the given category, and the GLS (Count) indicates the number of annotated GLS GWAS hits in a given category. A proportion z-test was used to test the abundance of genes in a given category relative to the overall abundance predicted in the maize genome. SE: standard error.

### Disease QTL confirmation and pleiotropic loci

The three most significant QTL based on the predictive model, *qGLS1.04*, *qGLS2.09*, *qGLS4.05*, were confirmed using near-isogenic line pairs (NILs) extracted from heterogeneous inbred families (HIFs; [44]) (Table 2). Two of these QTL for GLS were previously reported, while *qGLS1.04* is reported here for the first time. The families were composed of RIL that had been selfed for seven generations. Lines of the original S_5_ generation that were heterozygous at one of the three loci of interest were identified. Corresponding S_7_ lines were genotyped. For each HIF, at least six lines resulting from independent recombination events (three with the B73 allele and three with the alternate allele) were identified for further analysis of the three loci of interest.

**Fig 3:**
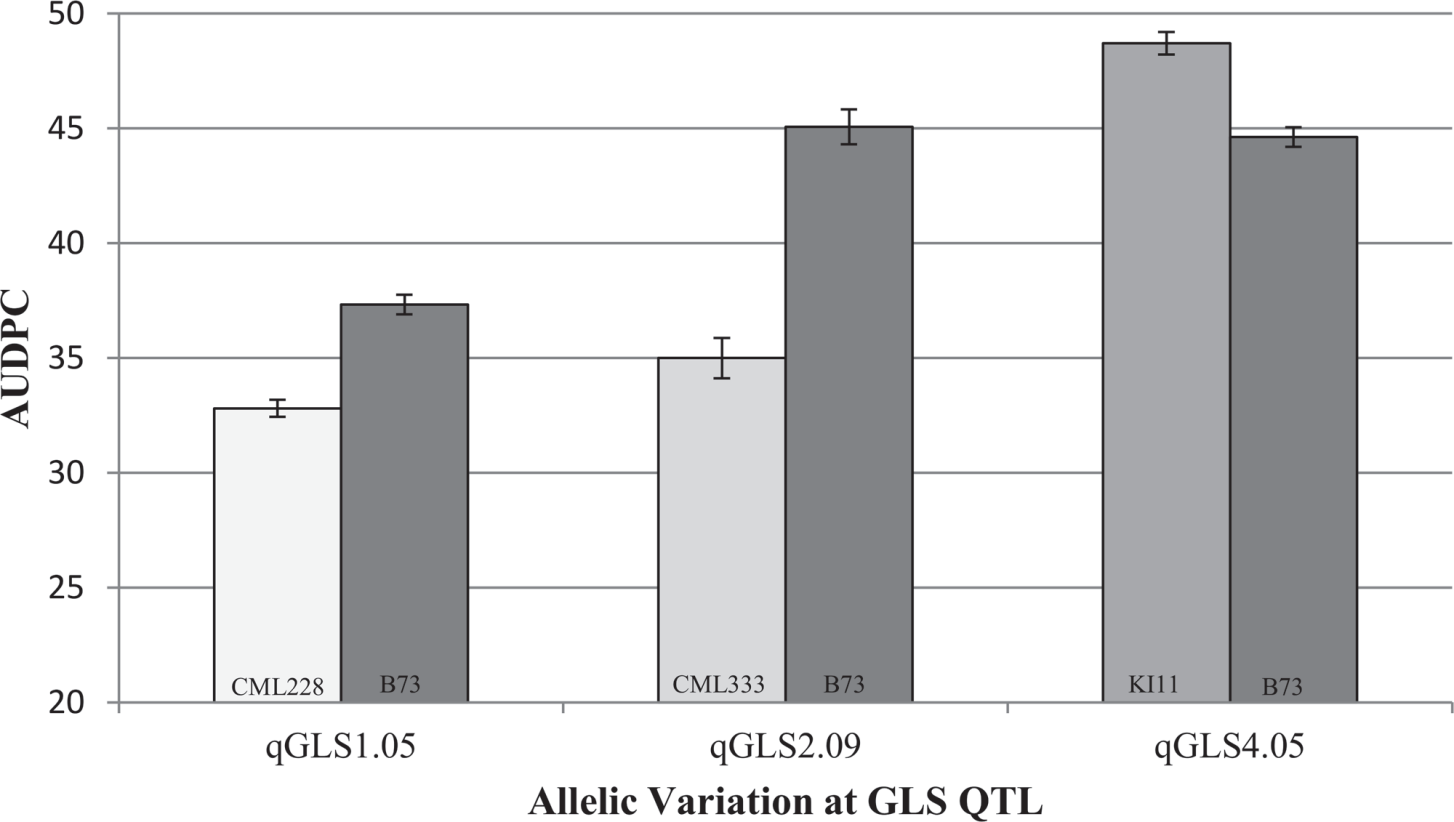
Confirmation of three disease quantitative trait loci using near isogenic lines. Disease development [area under the disease progress curve (AUDPC)]is indicated among heterogeneous inbred family lines across three gray leaf spot quantitative trait loci.

The observed levels of disease were significantly different among the NILs for the B73 and other parent alleles (Fig. 3; *p*<0.05). The observed allelic effects were higher than those expected based on model predictions. NILs with the CML228 allele at *qGLS1.04* exhibited an average of 12% less disease compared to NILs carrying the B73 allele at the same locus, while the model predicted a 2.5% disease reduction. Lines with the CML333 allele at *qGLS2.09* exhibited an average of 22% less disease while the model predicted a 5.2% reduction. Finally, lines with the KI11 allele at *qGLS4.05* exhibited an average disease increase of 8.4% relative to lines with the B73 allele at the same locus, while the model predicted a 5.1% increase in disease.

Buckler et al. [45] previously identified flowering time QTL using the NAM population. Days to anthesis (DTA) data from that study (obtained from Aurora, NY) as well as from the present study were analyzed in relation to GLS disease progress data. A quadratic relationship was identified when the DTA data from Buckler et al. [45] were associated with disease progress collected in Blacksburg, while the relationship between DTA and disease progress in Blacksburg was linear. A linear relationship is preferred for general linear modeling, so the only DTA dataset used in this study was collected from the Blacksburg location. Ten of the GLS QTL had confidence intervals that overlapped with those of DTA QTL (Table 2). The DTA allelic effects were predicted to be significant at *qGLS1.04*_CML228_ (*p* = 0.0402) and *qGLS4.05*_KI11_ (*p* = 0.0244). Although DTA differences were significant among the NILs (*p* = 0.0263), the LSM differences in disease between the two alleles at *qGLS4.05*_KI11_ did not change when DTA was removed from the model.

**Fig 4:**
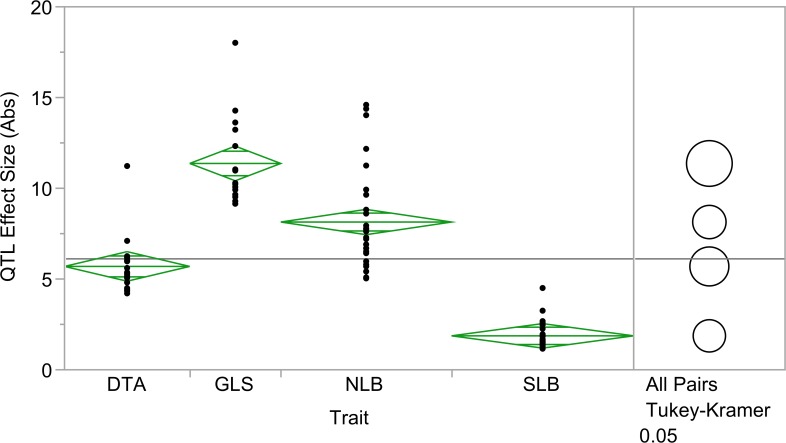
Absolute value of quantitative trait loci (QTL) effect size across three diseases and flowering time. Effect sizes of QTL for gray leaf spot, northern leaf blight, southern leaf blight and Blacksburg specific days to anthesis were significantly different (*p* = 6.33x10^–30^).

In addition to DTA, QTL for northern leaf blight (NLB) and southern leaf blight (SLB) were previously identified using the NAM population [40,46]. The QTL count and absolute value of the QTL effect size were analyzed across GLS, NLB, SLB and DTA. There were significant differences in QTL effect size across all four traits (Fig. 4; *p* = 6.33x10^–30^). Fewer, significantly larger effect QTL were identified for GLS than for NLB and SLB. NLB and SLB QTL counts were similar, but effect sizes for SLB were significantly smaller. There was evidence of pleiotropy at several loci, based on correlations among allele effects for the different diseases. Allelic effects of GLS QTL were positively correlated with those of NLB QTL at six loci (Table 2; *p*<0.05). GLS QTL were negatively associated with allelic effects of SLB QTL at one locus (Table 2; *p*<0.05).

### *qGLS1.04*_CML228_: fine-mapping and flavin-monooxygenase expression

NIL pairs were developed for the *qGLS1.04*_CML228_ locus. The allelic effect of the locus on GLS disease progress was determined, as noted above, and the locus was subsequently fine-mapped using derivatives of the same HIF population. The QTL interval spanned from 56,747,253 Mb to 83,780,725 Mb. The estimated recombination rate at this locus was 0.236 cM/Mb. The QTL was fine-mapped to two intervals of 77,242,690 to 83,780,725 Mb and 88,849,284 to 94,085,195 Mb, referred to as *qGLS1.04_1* and *qGLS1.04_2* (Table 4). The *qGLS1.04_1* interval contained 99 genes based on version 2 of the maize genome (www.maizesequence.org), while the *qGLS1.04_2* interval contained 51 genes. The genes within these regions were functionally annotated using BLAST. Both intervals in the 1.04 region were observed to have a high density of defense response (DR) genes. Genes implicated in detoxification constituted 13% of genes at the *qGLS1.04_1* locus and 9.8% at the *qGLS1.04_2* locus. Proportion tests revealed that putative glutathione-S-transferase genes were significantly more numerous than expected (Table 5).

**Table 4:** Markers and associated p-values from the qGLS1.04 fine-mapping analysis.

Marker	Chromosome	Location	p-value
PZA03168.5	1	51514741	0.808
PZA01267.3	1	77242690	0.5963
PZA00752.1	1	82019775	8.687E-08
PZA01135.1	1	83780725	0.1703
PZE0188095678	1	88095678	0.2202
PZB01235.4	1	93909140	0.0007
PZE0194085195	1	94085195	0.3395
PZA02750.3	1	101421637	0.549

**Table 5:** Summary statistics for detoxification-related genes underlying qGLS1.04.

Category	Genome (count)	qGLS1.04 (count)	SE	z-value	p-value
1-deoxy-D-xylulose 5-phosphate synthase	4	2	0.058	-0.229	0.409
Glutathione-s-transferase	573	11	0.031	-2.100	0.018
Oxidoreductase	214	4	0.031	-0.751	0.226
Phytoene synthase	4	4	0.081	-0.328	0.372

Summary statistics for functionally annotated and categorized genes within the qGLS1.04 quantitative trait loci hypothesized as putative detoxification-related genes.

SE = standard error.

**Fig 5:**
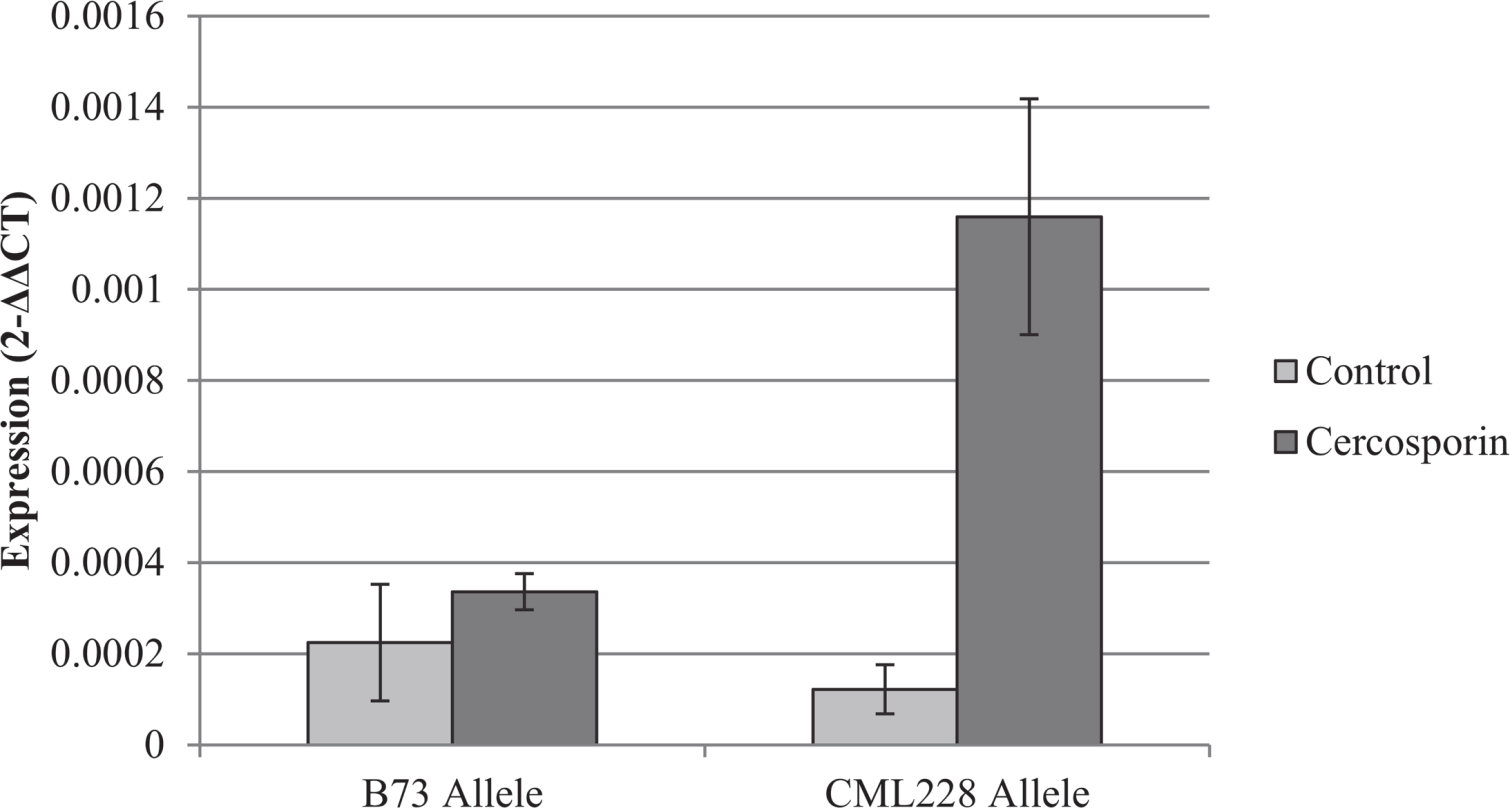
Analysis of flavin-monooxygenase expression at qGLS1.04. Expression differences were detected for the putative flavin-monooxygenase among heterogeneous inbred family lines segregating at *qGLS1.04* for the B73 or CML228 maize alleles. The heterogenous inbred family lines were treated with cercosporin or the acetone control on either side of the maize leaf midrib. There was a significant difference between the CML228 samples treated with cercosporin and the other samples in the experiment (*p* = 0.0012).

NILs were treated with cercosporin to test for DR genes playing a role in disease resistance. Expression was up-regulated 3.4-fold for GRMZM2G425719, a putative flavin-monooxygenase (FMO; Fig. 5). Up-regulation of other DR genes in the regions was not detected. Forty-four polymorphisms within the FMO were identified using HMPv2. Twenty of these polymorphisms were identified in the promoter region of the gene. G to A substitution was identified using a plant promoter algorithm to detect functional changes between the B73 and CML228 promoter regions. This change led to the detection of a putative functional TATA box within the CML228 allele. No significant difference was detected in carotenoid levels among the treated NILs.

### *qGLS2.09*_CML333_ and *qGLS4.05*_KI11_: fine-mapping

NIL pairs developed to be 99% isogenic were used to confirm and further fine-map *qGLS2.09*_CML333_ and *qGLS4.05*_KI11_. The fine-mapping population for *qGLS2.09*_CML333_ had poor seed set and was susceptible to drought stress. Only 375 plants survived from the 2450 kernels planted for fine-mapping during the 2011 field season. Irrigation needs were more closely monitored in 2012 to ensure higher survival rates (> 95%). The GLS QTL interval was reduced from 13 Mb to 4 Mb. This region had a recombination frequency of 0.908 cM/Mb and was predicted to contain 290 protein-coding genes.

*qGLS4.05*_KI11_ was initially considered an attractive target for fine-mapping because of its maximum LOD score of 44. The breakpoint density of this centromeric QTL could not be increased, however, due to the low recombination frequency of 0.05cM/Mb. This region had an estimated size of 140 Mb and contained as many as 7,500 protein-coding genes.

### Loci affecting inter-vein distance and conidiophore development

**Fig 6:**
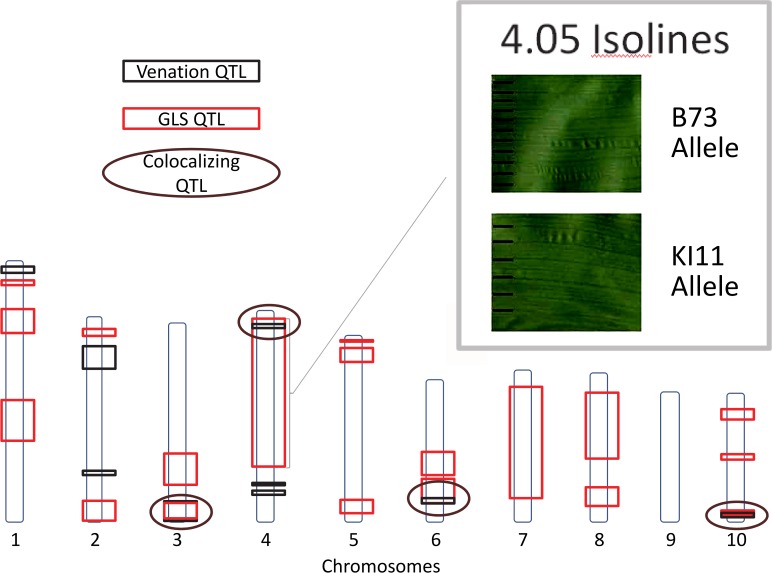
Gray leaf spot (GLS) and venation quantitative trait loci. Quantitative trait loci for GLS and inter-vein distance (IVD) are indicated. Circles designate co-localizing areas. Scanned leaf images (upper-right corner) demonstrate the difference between IVD from maize leaves with B73 and KI11 alleles at *qGLS4.05*.

Scanned images of GLS infection on maize leaves were analyzed using image analysis software. The distance between three major and three minor veins was measured for each leaf sample at the widest point of the leaf blade. A significant positive relationship between disease development (AUDPC) and the distance between major veins (IVD) was detected in the NAM population (*p*<0.0001). A significant relationship was not observed between minor vein distance and disease development. The observation that narrow IVD was associated with lower disease levels supported the hypothesis that this morphological trait could influence disease progression [26], and further suggested that QTL for disease and IVD might co-localize. To test this hypothesis, loci affecting IVD were analyzed using a model selection approach. Nine markers, located on six of the 10 chromosomes, were found to be associated with IVD. Similar to the disease QTL, effect sizes for IVD QTL across parental lines varied at each locus, and there were allelic effects significantly above and below the effect of the B73 allele. QTL for IVD and disease development co-localized at four intervals (Fig. 6). One of these QTL co-localized with *qGLS4.05*_KI11_. NILs for this locus were planted in the greenhouse and the average distance between veins was measured. A significant difference was detected between NILs containing the KI11 allele and those containing the B73 allele at the 4.05 locus (*p*<0.05).

**Fig 7:**
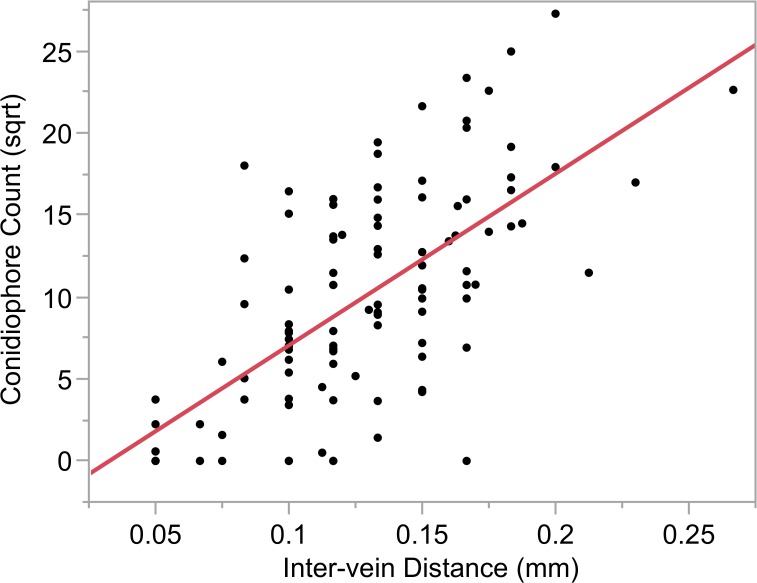
Relationship between conidiophore count and intervein distance. Significant relationship was detected between inter-vein distance and square root (sqrt) of the conidiophore counts (*r* ^*2*^ = 0.43; *p*<0.0001).

To assess the epidemiological relevance of narrow IVD, lesion parameters and conidiophore counts were collected from 2011 lesion samples. The conidiophore counts and IVDs for NAM parental lines were compared (Fig. 7). IVD accounted for 46% of variation in conidiophore count. A significant positive correlation was detected between IVD and the number of conidiophores per lesion (*p* = 2.34x10^–14^). This correlation suggests that the smaller the distance between the major veins, the lower the conidiophore count. IVD remained significant in a model predicting conidiophore count, with lesion length, length*IVD and pedigree as additional fixed effects (Table 6). There was a strong relationship between pedigree and IVD, such that IVD became less significant in a model with pedigree (*p*<0.0001). Lesion length and IVD were entered into the model to determine the effect of IVD when conidiophore variation attributed to length was taken into account. In this case, IVD accounted for 41% of the variation for conidiophore count.

**Table 6:** Model results with conidiophore count (square root) as the response variable and lesion parameter predictors.

Model	Source	DF	Sum of Squares	F Ratio	Prob > F	SS/TSS
1	Pedigree	21	964	6	2.03x10^−9^	0.5906
1	Length	1	566	73	5.83x10^−13^	0.3467
1	IVD	1	47	6	0.0159	0.0287
1	Length*IVD	1	55	7	0.0089	0.0340
2	Length	1	929	57	1.70x10^−11^	0.5898
2	IVD	1	646	40	7.16x10^−9^	0.4102

IVD = intervein distance

DF = degrees of freedom

Prob = probability

SS = sum of squares

TSS = total sum of squares.

## Discussion

In this study, seven novel GLS disease resistance loci were identified by nested association mapping in a diverse sample of maize germplasm. Another nine loci were identified that co-localized with GLS QTL previously identified from other studies (Table 2); seven of these were more precisely localized in this study than in previous analyses. Other QTL (e.g., *qGLS4.05*_KI11_) were not finely resolved due to the low recombination rates in the intervals. McMullen et al. [23] identified heavy segregation distortion on chromosome 4 within the B73 x KI11 family, and found that this region had a significantly greater proportion of B73 alleles than KI11 alleles. All QTL mapped by Maroof et al. [16] were identified using the NAM strategy. Our study was performed on the same field site over a decade later and, although it appears that the pathogen population had changed from one with both *Cercospora zeae-maydis* and *C*. *zeina* to one dominated by *C*. *zeae-maydis*, similar QTL and presumably similar plant resistance mechanisms appear to function.

Evidence was obtained for pleiotropic responses across diseases. Several loci appeared to condition resistance to both GLS and NLB; six of the 16 QTL showing positively correlated allelic effects. A locus may condition resistance to one or more diseases while acting as a susceptibility factor for another disease. For example, GLS parental allelic effects at *qGLS3.06* were negatively associated with SLB effects while positively associated with NLB.

Heritability of GLS resistance was high across the NAM population, and the QTL effects were generally large. Similar heritability estimates have been reported for other foliar diseases scored across the NAM population [40,46], despite differences in disease and rating methodology. For the three loci tested both in the NAM and in NILs, the estimated allelic effects were much larger based on NILs. The BLUPs of disease index ratings reduced the estimated variance, and allelic effect estimates were further deflated when maturity was taken into account. There were fewer QTL with larger effect estimates for GLS than for NLB and SLB, although each of these diseases was scored in the one location across three sequential field seasons. This may reflect the more recent emergence of GLS; it is possible that the pathogen population has had less time to co-evolve to partially overcome the resistance QTL compared to NLB and SLB. Similarly, the shorter time since emergence of the disease may mean that breeders have had less opportunity to accumulate minor resistance factors in maize germplasm.

In the NAM analyses of SLB and NLB, no interactions among disease QTL were detected [40,46]. Here we report interactions involving loci not directly contributing to resistance. In other systems, interactions have been reported to exist among functionally similar genes, often those that act in the same pathway [47] and can be elucidated using RNA interference methodologies [48], among others. Without further fine mapping, there were too many genes underlying the interacting loci to allow us to speculate on the pathways involved.

This study revealed a relationship between leaf structure (the distance between major veins) and GLS disease development. The 4.05 locus had pleiotropic effects on both disease and IVD. There was a positive correlation between IVD and disease development, suggesting that IVD-based lesion restriction may serve as a host resistance mechanism. Tropical germplasm tended to have greater IVD than temperate germplasm among the inbreds observed. These findings suggest that a lesion on an inbred with narrow veins produces fewer conidiophores than a lesion on an inbred with wide veins, resulting in reduced production of inoculum. The effect of reduced conidiophore production would be compounded across multiple reproductive cycles within one season, because *Cercospora zeae-maydis* is a polycyclic pathogen. Shorter IVD may decrease fungal reproduction via reduced lesion size (lower area available for fungal nourishment) and may also have indirect effects on fungal development via other morphological traits such as stomatal density (this could influence conidial production). A leaf width QTL identified in bin 4.05 [49] co-localized with GLS and venation QTL found in this study. Correlated effects of IVD and leaf width might influence yield through effects on photosynthetic capacity and/or harvest index. The pleiotropic effects of morphological phenotypes influencing disease and other traits merit greater investigation.

*qGLS1.04*, which was identified for the first time in this study, was validated and fine-mapped. Fine-mapping this locus provides breeders with markers that are closely linked to gene(s) conditioning the disease resistance phenotype. The novel QTL interval was fine-mapped from an initial interval of 27.0 Mb to two intervals of 6.5 Mb and 5.2 Mb, suggesting that multiple genes may underlie original QTL identified by NAM. Similar QTL fractionation has been identified in other quantitative disease resistance studies [50–52]. Increased marker density may help resolve one QTL into two (or more) if there is sufficient recombination in the region among the NAM founders.

Treatment of the *qGLS1.04* NILs with cercosporin increased expression of a putative flavin-monooxygenase (FMO) gene. FMOs are a family of oxidoreductases that have been previously implicated in disease resistance. For example, FMOs play a role in glucosinolate production [53,54]. Glucosinolates are a class of secondary metabolites noted for their role in fungal disease resistance among the brassicaceae [55]. Mishina and Zeier [56] determined that FMOs play a role in biologically-induced systemic acquired resistance. Oxidoreductases have also been implicated in the degradation of cercosporin into non-toxic xanosporic acid. Taylor et al. [57] identified mutant strains of *Xanthomonas campestris* that could no longer degrade cercosporin, while its wild-type progenitor had this ability. All mutants in the Taylor et al. [57] study could be complemented with a genomic clone with homologous sequence to a transcriptional regulator and an oxidoreductase. The mutants had point mutations in the oxidoreductase and not in the regulator, but expression of both was necessary for complementation [57]. The putative FMO may play a role in cercosporin degradation, because it was upregulated by cercosporin application. In future studies, this might be tested by transforming *X*. *campestris* wild-type strains with sequences coding for the putatative FMO to verify the gene’s involvement in cercosporin degradation.

Our findings lend preliminary support to the hypothesis that genes underlying quantitative disease resistance are involved in mitigating the effects of microbial compounds deployed during the pathogenesis process [26]. Other studies have implicated genes related to detoxification of microbial compounds in resistance to GLS. In a multivariate analysis of resistance to GLS, NLB and SLB, Wisser et al. [58] identified a significant association across all three diseases with a glutathione-S-transferase. This gene lies within the confidence interval of *qGLS7.03* and is considered responsible for reducing oxidative stress and detoxification of microbial compounds. The development of a *qGLS1.04* FMO mutant for disease resistance studies would provide more evidence needed to validate the findings. Multiple genes are likely to be involved in conferring resistance at *qGLS1.04*, because there are multiple fine-mapping intervals underlying resistance at this locus.

The results of this study provide a more thorough and higher-resolution understanding of the genetic architecture of GLS resistance and provide initial support for the hypothesis that structural and detoxification mechanisms underlie quantitative resistance to GLS of maize. Plant breeding decisions regarding development and deployment of resistance will be improved with better understanding of the mechanisms underlying quantitative disease resistance, especially as the mechanisms relate to important agronomic traits such as leaf anatomy and days to maturity.

## Materials and Methods

### Plant materials and field site

The NAM population was developed by the Maize Diversity Project as a public resource (www.panzea.org) [23]. The NAM population consists of 25 recombinant inbred line (RIL) families derived from crossing each of 25 diverse maize lines with a single reference parent (B73) [23]. The majority of the NAM population (3,678 lines) was planted at Virginia Polytechnic Institute’s Whitethorne Research Farm located in Blacksburg, VA. In addition, 150 lines of the Intermated B73 x Mo17 population (IBM) were included. Line selection was based on seed availability and predicted experimental power [59]. Three replications of the populations were planted in 2008, 2009 and 2010 (one replication/year). Sixteen kernels were planted in 2.4-m long rows with 0.3-m row spacing for each line. The population was arranged in an incomplete block design, augmented by blocks that contained two parental checks. The blocks were arranged by families within the NAM.

The Whitethorne Research Farm was chosen for high and consistent disease pressure that is routinely observed from natural inoculum. Maize had been continuously planted in the field under no-till conditions since 1985. The field had been manually inoculated for three seasons (1985, 1986, and 1987) prior to dependence on natural inoculum. The isolates originally used to inoculate the field (VA-1, VA-2 and VA-3) were collected from maize fields located in Montgomery County and Wythe County, Virginia, in 1985. These isolates were initially identified as only *Cercospora zeae-maydis*, but were later found to be a mixture of *C*. *zeae-maydis* I and *C*. *zeae-maydis* II. *C*. *zeae-maydis* II has subsequently been reclassified as *C*. *zeina* [4]. Sporulation of *C*. *zeae-maydis* and *C*. *zeina* on the residues from the previous seasons likely provided primary inoculum for disease development on the maize plants. Diseased leaf samples were collected at random from a subset of the parental lines at the conclusion of the 2009 and 2010 seasons. For each year, 25–50 isolations were made. Isolates were identified at the genus level based on conidial morphology and identified at the species level using colony traits when grown on potato dextrose agar. Isolates producing the characteristic purple halo (cercosporin) and exhibiting faster colony growth were inferred to be *C*. *zeae-maydis* [60]. These tests have been extensively compared with molecular typing in our laboratory and found to be reliable for distinguishing the two species [60]. The majority of samples (90–95%) were identified as *C*. *zeae-maydis*.

### Phenotypic assessment

A disease rating methodology was modified to include increments of 0.25 on a 0–5 scale [16]. Using this 21-point scale, each line was scored three times at seven-day intervals. Ratings were made after flowering time (dehiscence) for most lines (S2 Table, S1 Dataset). Area under the disease progress curve (AUDPC) was calculated from the resulting disease scores for each line. Days to anthesis (DTA) data, defined as days from planting to anthesis of 50% of row plants, were also collected (S2 Dataset).

Scanned images of GLS infection on maize leaves were collected in 2009 and 2010. Ear leaves were sampled from each RIL three times at 10-d intervals. These leaves were collected after flowering time, when the GLS symptoms began to develop. Leaves were transferred to the lab on the day of sampling, mounted on a white sheet of paper, and scanned with the corresponding identification. We developed digital image analysis software to analyze scanned leaf images. The software measured the distance between three major and three minor veins on each leaf sample, and the values were averaged for each NAM line. Venation structure was measured at the widest part of the leaf right above the midrib, because leaves within the population varied in size and shape (S3 Table, S3 Dataset). The software also measured the dimensions of each lesion and the number of lesions within a defined area.

Lesion samples were collected in 2011 from the parents of the NAM population to assess sporulation in relation to lesion size. Samples were boiled in 1 M KOH and then rinsed with fresh, sterile, autoclaved water. The rinse was repeated several times over a two week period, resulting in cleared leaf samples that were devoid of chlorophyll and other pigments. Each sample was then mounted on a slide and examined under a light microscope. Conidiophores within the lesions were counted using a manual counter and the computer-projected image (S4 Table). Distance between the major veins was measured using a standard metric ruler.

### Analysis

Best linear unbiased predictions (BLUPs) of the GLS disease scores were acquired using ASReml3 statistical software as described by Poland et al. [40]. BLUPs extracted from this model were used to calculate AUDPC, which was used as the response variable in the PROC GLMSELECT stepwise selection procedure in SAS 9.3 (SAS Institute Inc., Cary, NC). Covariates included family and DTA. Common-parent-specific markers (*n* = 1,106) were also used as predictor variables with a selection threshold (*p* = 1 x 10^–4^). QTL mapping was undertaken using a general linear modeling approach with PROC GLM in SAS 9.3 (SAS Institute Inc., Cary, NC).

Markers selected as significant contributors in the stepwise selection model were identified as QTL, while the associated model estimates were considered allelic effects (S5 Table). The sum of allelic effects in the parental lines was compared to the parental phenotypic BLUP in order to determine the percent phenotypic variation explained by the QTL [40]. A general linear model (GLM) was constructed using the selected markers associated with the QTL as predictors, as well as family and flowering time as covariates. Variations of this model were used to construct confidence intervals, identify interactions, and determine least squared means (LSM) of AUDPC for a given allelic effect of a given QTL (S6 Table). The variance components of family and RIL were used to describe genetic variance for the NAM population. Heritabilities on an individual plot basis and on a line mean basis were estimated for the entire NAM population as described in Hung et al. [43]. A mid-parent offspring regression was used to predict narrow sense heritability. Confidence intervals were identified by removing one marker at a time from the full linear model and inserting the associated flanking markers individually until the flanking markers failed to significantly describe the response variable at *p*<0.0001 [40]. QTL-QTL interactions and interactions between QTL-associated markers and non-significant markers were included in the GLM to identify significant interactions (*p*<0.00001). Pleiotropic loci and alleles affecting both flowering time and disease development were identified by substituting DTA for AUDPC as the response variable in the GLM described above.

Residuals for each chromosome were extracted from the GLM by removing physically linked markers, one linkage group at a time, from the model (S4 Dataset). These residuals were submitted to NAM-Genome Wide Association Study (NAM-GWAS) at BioHPC for genome-wide single nucleotide polymorphisms (SNP) association using the bootstrap regression analysis option [49]. Significant GWAS hits were functionally annotated using basic local alignment search tool (BLAST). The sequence of each gene associated with a significant GWAS hit was pulled from the maize B73 reference genome (V2). The gene sequences were then populated in the gene ontology BLAST search and run using the default settings of 0.1 Expect threshold, 50 Max # alignments and “all” selected for gene product types, data sources, species, ontology and evidence code (AmiGO 1.8; http://amigo1.geneontology.org/cgi-bin/amigo/blast.cgi?). These annotations were categorized into biological groups, which included: structural components, secondary metabolites, primary metabolites, genes previously implicated in pathogen defense, membrane transporter, development, cellular energy, cell signaling, and cell cycle. The genes on the inter-pro hit list (www.maizesequence.org) were also categorized into the same groups. A proportion z-test was used to test the abundance of genes in a given category relative to the overall abundance predicted in the maize genome.

### Heterogenous inbred family development, QTL confirmation & fine-mapping

Specific RILs composing the NAM were selected for heterogenous inbred family (HIF) development [44]. Forty-three lines met the criteria of segregating at one of three QTL and being fixed at all other significant QTL. The three QTL of interest were those that corresponded to the three markers that most significantly described GLS AUDPC. These lines were selected from six subpopulations of the NAM based on the predicted effect of the allele at the specific locus on disease development. The derived lines contrasting for each QTL are hereafter referred to as near isogenic lines (NILs).

The selected lines were selfed and then genotyped in 2009 at Cornell University’s Musgrave Research Farm in Aurora, NY. Heterozygous plants at the loci of interest were selfed in 2010 winter nursery. Fixed lines were selected for random placement in one of six pedigree-based Latin square designs on Whitethorne Farm in summer 2010. These lines were genotyped and scored using the disease rating methodology described above. Lines within the same HIF were analyzed for significant phenotype and genotype association at loci of interest. Heterozygous lines in the region implicated in disease resistance were again advanced in a winter field season, genotyped and planted in an incomplete block design that included both heterozygous and fixed lines in the 2011 and 2012 field seasons. A total of 1,750 and 6,175 plants were screened and genotyped in the 2011 and 2012 field seasons, respectively, in order to increase statistical power and to increase the likelihood of identifying an advantageous recombination breakpoint.

Experimental units (rows in 2010 and individual plants in 2011 and 2012) were scored three times for disease at seven day intervals, which were used to calculate AUDPC. Flowering time data were also collected for each experimental unit in 2010 and 2011. Experimental units with like haplotypes were analyzed together. Like haplotypes were identified as having the same genotypes flanking the segregating regions. The experimental units were analyzed using PROC GLM in SAS 9.3 (SAS Institute Inc., Cary, NC). Disease development (AUDPC) was the response variable and predictors were the genotypes within the QTL confidence or fine-mapping interval. False discovery rate (FDR) was calculated independently for each year based on the number of markers analyzed and used as a threshold for significant associations between the disease phenotypes and marker genotypes.

### Venation confirmation at the 4.05 locus

One of the venation QTL co-localized with *qGLS4.05*_KI11_ and with a fine-mapping population already developed for this locus, the NILs were planted in the greenhouse to measure average distance between veins. The experiment was set up in a complete randomized block with 10 replications of 7 HIF lines. The plants were grown to just beyond the R1stage when all lines had reached the point of anthesis. As with the scanning protocol described above, leaves were transferred to the lab on the day of sampling, mounted on a white sheet of paper, and scanned with the corresponding identification. The software measured the distance between three major and three minor veins and the values were averaged for each NAM line. Venation structure was measured at the widest part of the leaf right above the midrib, because leaves within the population varied in size and shape. A t-test was used to detect a significant difference between the isolines.

### Functional annotation of NAM-GWAS and genes within the fine-mapping interval

A list of genes within the *qGLS1.04* fine-mapping intervals (1:77,242,690 to 1:83,780,725 Mb and 1:88,849,284 to 1:94,085,195 Mb) was exported from the maize genome browser (www.maizesequence.org). These genes were functionally annotated using BLAST and compared to the maize top 500 inter-pro hit list. An initial inspection indicated that the region was rich in detoxification-related genes. The z-test is used to determine if the hypothesized population proportion is significantly different from the sampled proportions and as such, it was used to evaluate the genome abundance of detoxification-related (DR) genes compared to the observed sample differences. Specific gene families identified by sequence inspection were tested for enrichment using the z-test.

### Cercosporin treatment of 1.04 isolines

Twenty-four near isogenic lines (F6:8NILs) developed using the HIF strategy were grown in the greenhouse under standard maize growing conditions. Lines were organized in a randomized complete block design (4 blocks). Three of the six plants in each block contained the susceptible B73 allele, and the other three contained the resistant CML228 allele. Different lines in the same HIF family were used to account for residual background effect resulting from regions that may still have been segregating (estimated at less than 0.5%).

At flowering time, two ear leaves on each plant were treated with 0.1 ml of 100 μM cercosporin in acetone and an acetone control using a procedure modified from Batchvarova et al. [61]. Treatment and control were infiltrated using a needleless syringe. The control and cercosporin treatments were applied to the same leaf on either side of the midrib. Plants were placed in constant light for 24 h in order to activate the cercosporin. After this time period, 10 leaf punches of 6-mm diameter were collected directly around each treated site using a paper punch (2 controls and 2 treatments per plant). Samples collected from the lower ear leaf were used for carotenoid detection and samples from the upper ear leaf were used for expression analysis.

Carotenoids were extracted and measured from maize leaf tissue using modified procedures based on those of Alba et al. (2005) and Bushway (1986). Under low light conditions, 100 mg of pre-weighed tissue was homogenized with 50 μl of a 0.3% MgCO_3_ solution (w:v) and 300 μl of tetrahydrofuran (THF). Homogenization was repeated after addition of 300 μl of 0.5% butylated hydroxyl-toluene/methanol (w:v). An additional 600 μl of THF was added to the extract, which was then filtered. To the filtered extract, 50 μl of 25% NaCl and 600 μl of petroleum ether were added and the sample was vortexed well. The upper phase was dried down and 500 μL HPLC grade ethyl acetate was added in preparation for the column; this was mixed well and filtered. A sample of the extract was added to a YMC C_30_ column for reverse-phase high performance liquid chromatography (RP-HPLC).

Real-time quantitative PCR (qRT-PCR) was performed on cercosporin- and control-treated HIFs to test for expression differences across genes hypothesized to play a role in cercosporin detoxification. Total RNA was purified from maize leaf tissue using the RNEasy Mini Kit (Qiagen). The same kit was used for DNase I digestion. The cDNA was prepared using the SuperScript III First-Strand Synthesis SuperMix for qRT-PCR (Invitrogen). Primer pairs were designed for candidate detoxification-related genes (Table 7) and used for RT-PCR with Power SYBR Green PCR Master Mix (Invitrogen). Data were analyzed using the Comparative CT method [62] (S7 Table).

**Table 7:** Primer sequences used for qGLS1.04_CML228_ expression tests.

Start	End	Functional Annotation	Primer Sequence
81777188	81778493	Glutathione Transferase30	CCTCTGCGCGTGTATCTCGTCG
81777188	81778493		CCTGCACCTCAGGTCCCTCCA
82187887	82190028	Bronze (BZ)-R gene	TGCACCTGCCAGATCCTGTCCA
82187887	82190028		GACGGCCGGGGGATGGGATT
82213773	82215225	Chloroplast Phytoene Synthase (Y1)	CACACAGCCGCCTCTCACCG
82213773	82215225		GCTGGATGCTGGAAGGGTGCC
82578863	82582627	Chalcone Synthase (C2)	ACGTTCCACCACACCCACACG
82578863	82582627		CCAGATGGCTCAGGTAACCTCGATT
83400264	83404419	Glyceraldehyde-3-Phosphate Dehydrogenase (GPC2)	AGAGCACGTGGACGTGGATCTGATT
83400264	83404419		AACTTGGCAAAAAGACGGTTGCCCA
83431960	83433879	Chloroplast Phytoene Synthase (Y1)	GCAAACGGGGCCCGGCATC
83431960	83433879		TGGCCAGAATCGGACTCGAGCG
84671535	84674990	Chloroplast Phytoene Synthase (Y1)	CGTTCTCGCCCAGTCGCACC
84671535	84674990		TGCAGACCAATCAGCTCCCAACA
87011117	87011985	Carotenoid Cleavage Dioxygenase 1 (CCD1)	AGCACAGGAGGATTCAGAGGCT
87011117	87011985		TGAGTGAATCAGCGAGGGATCCAA
92342987	92345452	Flavin-Monooxygenase (FMO)	CCGTCACGCCACCAATCCCC
92342987	92345452		GATGCCCACTGGAGCCACCG
96688490	96691067	Zeta-Carotene Desaturase (ZDS1)	AGCCCATGAAGCGAGCAACCC
96688490	96691067		TGCAATTGGCGTCGTATGAAGTGA

List of gene locations on chromosome one and primer sequences used for expression tests on heterogeneous inbred family lines treated with cercosporin.

### Flavin-monooxygenase single nucleotide polymorphisms

SNPs between B73 and CML228 within the putative flavin-monooxygenase gene were identified using maize Haplotype Map Version 2 (HMPv2; www.panzea.org). The B73 and CML228 gene sequences were developed by aligning SNP calls with the sequence provided by the Maize Genome Sequence Consortium. The B73 SNP calls from HMPv2 matched the B73 reference genome. The promoter region sequence for the B73 and CML228 alleles was analyzed using plant promoter prediction software (Prediction of PLANT Promoters using RegSite Plant DB, Softberry Inc.) to detect putative functional differences in the promoter region between the parental lines.

## Supporting Information

S1 Table Genome wide association hits for resistance to gray leaf spot. Functionally annotated genome wide association hits. A 10 Kilobase window on either side of the GWA hit was screened for genes. Chr = Chromosome; BPP = Bootstrap posterior probability.
(DOCX)

S2 Table Raw gray leaf spot data collected on the nested association mapping population. (XLSX)

S3 Table Raw venation data collected on nested association mapping population. (XLSX)

S4 Table Inter-vein distance and associated conidiophore count for nested association mapping parental line leaf tissue samples. (XLSX)

S5 Table Quantitative trait loci effect estimates for days to anthesis, venation, northern leaf blight and southern leaf blight. (XLSX)

S6 Table Model predictions for the percent change in disease across parental alleles at each significant locus. (XLSX)

S7 Table Expression data for putative flavin-monooxygenase for near isogenic lines treated with cercosporin. (XLSX)

S1 Dataset Best linear unbiased predictors for gray leaf spot, northern leaf blight and southern leaf blight. (TXT)

S2 Dataset Best linear unbiased predictors for days to anthesis at the Blacksburg, VA location. (TXT)

S3 Dataset Best linear unbiased predictors for venation. (TXT)

S4 Dataset Gray leaf spot residuals used in the genome wide nested association analysis. (TXT)
