# Supplementary material for: Resistance to Gray Leaf Spot of Maize: Genetic Architecture and Mechanisms Elucidated through Nested Association Mapping and Near-Isogenic Line Analysis
Source: PLoS Genet. 2015 Mar 12;11(3):e1005045. doi: 10.1371/journal.pgen.1005045 (PMC4357430; doi:10.1371/journal.pgen.1005045)
Supplement: S1 Table — Functionally annotated genome wide association hits. A 10 Kilobase window on either side of the GWA hit was screened for genes. Chr = Chromosome; BPP = Bootstrap posterior probability. (DOCX) [file pgen.1005045.s001.docx]

| **Table S1. Genome wide association hits for resistance to gray leaf spot.** Functionally annotated genome wide association hits. A 10 Kilobase window on either side of the GWA hit was screened for genes. Chr=Chromosome; BPP=Bootstrap posterior probability. | | | | | | |
| --- | --- | --- | --- | --- | --- | --- |
| Chr | Marker Position | allele | effect | BPP | Gene | Functional Annotation |
| 1 | 2,030,770 | T/C | 0.62 | 7 | GRMZM2G164696 | Maize for beta 1 tubulin |
| 1 | 9,931,873 | C/G | 0.71 | 10 | GRMZM2G178571 | *Zea mays* 1-deoxy-D-xylulose 5-phosphate synthase 1 |
| 1 | 21,532,972 | G/A | -0.52 | 8 | GRMZM2G086604 | *Zea mays* retrotransposon Cinful-1 |
| 1 | 21,557,348 | G/T | 0.93 | 7 | Intergenic |  |
| 1 | 26,273,179 | C/T | 1.47 | 8 | Intergenic |  |
| 1 | 64,986,440 | C/G | -0.51 | 10 | GRMZM2G127181 | uncharacterized |
| 1 | 74,843,220 | T/C | -0.36 | 6 | Intergenic |  |
| 1 | 75,268,439 | G/A | -0.60 | 14 | Intergenic |  |
| 1 | 173,110,701 | G/A | -2.00 | 8 | Intergenic |  |
| 1 | 180,262,819 | C/T | -1.39 | 16 | Intergenic |  |
| 1 | 183,969,702 | G/T | -0.70 | 12 | GRMZM2G080746 | uncharacterized |
| 1 | 186,614,192 | T/C | -1.01 | 35 | AC205695.3_FG008 | uncharacterized |
| 1 | 187,927,685 | G/A | -1.87 | 26 | Intergenic |  |
| 1 | 198,970,183 | T/C | 0.74 | 7 | Intergenic |  |
| 1 | 199,579,946 | A/G | -0.85 | 7 | Intergenic |  |
| 1 | 201,295,476 | G/A | -0.63 | 12 | Intergenic |  |
| 1 | 207,120,463 | --/CC | -0.77 | 7 | GRMZM2G303157 | *Zea mays* T cytoplasm male sterility restorer factor 2 |
| 1 | 284,043,464 | G/A | 0.43 | 6 | Intergenic |  |
| 1 | 284,048,249 | C/A | 0.49 | 6 | GRMZM2G069772 | *Zea mays* rust resistance protein rp3-1 |
| 1 | 284,837,740 | A/G | 0.30 | 9 | Intergenic |  |
| 1 | 284,841,425 | G/T | 0.46 | 8 | Intergenic |  |
| 1 | 284,892,880 | A/C | 0.34 | 13 | Intergenic |  |
| 1 | 286,994,723 | G/A | 0.63 | 8 | GRMZM2G371210 | phytoene synthase (PSY1) |
| 1 | 293,630,563 | -------/GCCACAT | -2.93 | 7 | GRMZM2G068117 | *Zea mays* putative pol protein |
| 2 | 13,580,508 | C/G | -0.59 | 11 | cDNA |  |
| 2 | 17,397,612 | G/T | -0.66 | 7 | Intergenic |  |
| 2 | 17,422,032 | A/T | -0.75 | 8 | GRMZM2G057131 | rust resistance protein rp3-1 (rp3-1) |
| 2 | 20,029,416 | C/G | -0.66 | 10 | Intergenic |  |
| 2 | 20,639,672 | CTAGC/----- | -0.70 | 7 | Intergenic |  |
| 2 | 20,971,563 | C/A | -0.80 | 7 | Intergenic |  |
| 2 | 21,542,600 | G/C | -0.95 | 9 | GRMZM2G425211 | uncharacterized |
| 2 | 22,215,570 | C/A | -0.58 | 7 | GRMZM2G545802 | putative growth-regulating factor 1 |
| 2 | 172,704,846 | A/T | 0.46 | 7 | GRMZM2G000601 | ubiquitin-conjugating enzyme E2-17 kDa |
| 2 | 225,963,000 | C/A | -1.49 | 26 | Intergenic |  |
| 2 | 228,976,174 | C/A | -1.72 | 7 | Intergenic |  |
| 2 | 229,145,253 | G/A | -1.35 | 39 | GRMZM2G041642 | rust resistance protein rp3-1 (rp3-1) |
| 2 | 229,637,503 | C/G | -2.41 | 7 | Intergenic |  |
| 2 | 229,862,946 | T/G | -0.93 | 16 | GRMZM2G391164 | uncharacterized |
| 2 | 230,414,321 | C/T | -1.18 | 6 | GRMZM2G152258 | tropinone reductase 2 |
| 2 | 230,584,153 | A/G | -1.89 | 7 | GRMZM2G513206 | ramosa 2 (ra2) |
| 2 | 230,615,501 | T/G | -0.88 | 10 | GRMZM2G052403 | Tha8 (tha8) |
| 2 | 230,898,848 | T/A | -0.53 | 7 | GRMZM2G082302 | uncharacterized |
| 3 | 163,420,613 | G/A | 0.84 | 12 | Intergenic |  |
| 3 | 163,875,247 | T/G | 1.21 | 16 | Intergenic |  |
| 3 | 163,888,333 | G/A | 0.81 | 35 | Intergenic |  |
| 3 | 165,031,510 | T/C | 1.08 | 9 | Intergenic |  |
| 3 | 165,509,907 | G/T | 0.82 | 7 | GRMZM2G444541 | *Zea mays* cultivar B73 chloroplast |
| 3 | 165,792,180 | T/A | 0.84 | 16 | Intergenic |  |
| 3 | 167,576,843 | C/- | 0.57 | 7 | Intergenic |  |
| 3 | 202,841,878 | C/T | -0.70 | 43 | AC207628.4_FG011 | *Zea mays* see2a for putative legumain |
| 3 | 206,959,629 | C/G | -0.53 | 9 | GRMZM2G028568 | phytoene synthase (Y1) |
| 3 | 217,329,015 | G/A | 0.61 | 8 | Intergenic |  |
| 4 | 11,272,990 | C/T | -1.46 | 13 | GRMZM2G068330 | *Zea mays* protein dimerisation region containing protein |
| 4 | 12,987,147 | G/A | -1.19 | 6 | GRMZM2G124593 | uncharacterized |
| 4 | 39,437,101 | C/T | -0.49 | 22 | Intergenic |  |
| 4 | 93,594,993 | T/G | -1.28 | 28 | Intergenic |  |
| 4 | 137,271,558 | T/G | -0.84 | 11 | Intergenic |  |
| 4 | 140,210,546 | C/T | -1.66 | 81 | Intergenic |  |
| 4 | 143,945,947 | C/T | -0.57 | 33 | Intergenic |  |
| 4 | 147,941,545 | ---/GTC | 1.05 | 10 | Intergenic |  |
| 4 | 149,532,029 | A/T | -0.61 | 9 | Intergenic |  |
| 4 | 160,911,650 | C/A | -0.70 | 7 | Intergenic |  |
| 4 | 161,434,924 | G/A | -0.70 | 6 | GRMZM2G137696 | *Zea mays* discolored-1 (mutant allele dsc1-Ref::Mu1) |
| 4 | 245,875,432 | C/A | -0.87 | 11 | Intergenic |  |
| 5 | 4,244,452 | C/T | -0.33 | 12 | GRMZM2G007063 | *Zea mays* opaque-2 heterodimerizing protein 1b (ohp1b) |
| 5 | 4,294,169 | A/C | -0.52 | 12 | GRMZM5G801939 | uncharacterized |
| 5 | 4,669,956 | G/C | -0.52 | 11 | GRMZM2G027495 | *Zea mays* B73 acc oxidase (ACO35) |
| 5 | 6,369,752 | C/T | -1.26 | 7 | GRMZM2G176042 | uncharacterized |
| 5 | 6,418,770 | A/G | -1.01 | 10 | GRMZM2G077404 | *Zea mays* arginine N-methyltransferase 2 |
| 5 | 6,536,883 | G/C | -1.20 | 15 | GRMZM2G095185 | uncharacterized |
| 5 | 6,540,639 | A/T | -1.47 | 15 | GRMZM2G090432 | uncharacterized |
| 5 | 6,588,968 | A/T | -1.22 | 22 | Intergenic |  |
| 5 | 7,088,865 | A/G | -0.82 | 12 | Intergenic |  |
| 5 | 20,486,425 | --/TA | 0.77 | 6 | GRMZM2G080231 | uncharacterized |
| 5 | 21,974,701 | C/G | 0.81 | 9 | Intergenic |  |
| 5 | 25,824,240 | C/A | -1.00 | 13 | Intergenic |  |
| 5 | 40,275,106 | T/C | 0.93 | 16 | Intergenic |  |
| 5 | 56,244,216 | A/T | -1.03 | 6 | Intergenic |  |
| 5 | 181,353,895 | --/CT | 0.50 | 6 | Intergenic |  |
| 5 | 182,336,996 | C/T | 0.64 | 6 | Intergenic |  |
| 5 | 194,708,029 | C/G | 0.40 | 11 | GRMZM2G144097 | *Zea mays* folylpolyglutamate synthase (LOC100285702) |
| 5 | 197,017,506 | -/G | 0.57 | 7 | Intergenic |  |
| 5 | 204,100,563 | T/G | 0.59 | 26 | GRMZM2G029186 | uncharacterized |
| 5 | 205,473,574 | G/C | 0.82 | 22 | AC195458.3_FG001 | uncharacterized |
| 6 | 96,317,088 | T/C | 0.37 | 9 | Intergenic |  |
| 6 | 96,520,306 | TGT/--- | 0.58 | 9 | GRMZM2G472187 | uncharacterized |
| 6 | 97,120,550 | G/C | 0.97 | 11 | GRMZM2G131020 | *Zea mays* CUE domain containing protein (LOC100282879) |
| 6 | 104,876,576 | G/T | -1.08 | 6 | Intergenic |  |
| 6 | 108,329,244 | C/G | -1.11 | 9 | GRMZM2G701063 | *Zea mays* transcriptional activator |
| 6 | 109,996,701 | T/C | -0.55 | 11 | Intergenic |  |
| 6 | 110,193,339 | C/A | -0.76 | 9 | Intergenic |  |
| 6 | 110,249,748 | TC/-- | -0.66 | 11 | Intergenic |  |
| 6 | 129,925,381 | C/T | -0.42 | 12 | Intergenic |  |
| 6 | 138,425,664 | T/C | -0.42 | 6 | GRMZM2G117439 | uncharacterized |
| 6 | 143,322,264 | -----/GCTCG | -0.36 | 6 | Intergenic |  |
| 6 | 149,244,757 | T/C | -0.48 | 6 | Intergenic |  |
| 7 | 2,931,863 | G/C | -0.74 | 6 | Intergenic |  |
| 7 | 3,212,361 | -------/CTCGATT | -0.56 | 6 | Intergenic |  |
| 7 | 121,127,424 | ------/CTCCCA | -0.54 | 6 | Intergenic |  |
| 7 | 123,595,279 | G/A | -0.54 | 67 | GRMZM2G154752 | uncharacterized |
| 7 | 158,495,841 | C/A | 0.66 | 6 | Intergenic |  |
| 7 | 159,611,041 | -/A | -0.53 | 11 | GRMZM2G158452 | uncharacterized |
| 7 | 162,122,347 | T/A | -1.37 | 11 | Intergenic |  |
| 8 | 8,431,762 | T/A | 0.86 | 6 | Intergenic |  |
| 8 | 9,495,845 | C/T | 0.34 | 7 | GRMZM2G473485 | *Zea mays* rust resistance protein rp3-1 (rp3-1) |
| 8 | 13,658,348 | G/T | 1.02 | 6 | AC187393.3_FG001 | *Zea mays* heat shock factor-binding protein 1 (hsbp1) |
| 8 | 20,706,941 | G/A | -0.64 | 7 | GRMZM2G080588 | Z.mays GapC2 |
| 8 | 26,104,142 | -/G | -0.64 | 9 | GRMZM2G154221 | *Zea mays* T cytoplasm male sterility restorer factor 2 (rf2a) |
| 8 | 35,882,697 | G/C | -0.86 | 10 | Intergenic |  |
| 8 | 49,566,758 | G/A | -0.90 | 24 | Intergenic |  |
| 8 | 57,863,663 | G/A | -1.07 | 30 | Intergenic |  |
| 8 | 72,046,701 | ---/AAC | -0.77 | 7 | Intergenic |  |
| 8 | 105,554,753 | C/T | 0.80 | 6 | Intergenic |  |
| 8 | 141,605,883 | G/A | 1.08 | 8 | GRMZM2G015735 | *Zea mays* chloroplast phytoene synthase (Y1) |
| 8 | 146,531,554 | -/G | 0.77 | 6 | Intergenic |  |
| 8 | 149,501,067 | A/T | 0.71 | 6 | Intergenic |  |
| 8 | 154,091,270 | A/G | 1.29 | 8 | Intergenic |  |
| 8 | 158,597,202 | C/T | 0.42 | 23 | Intergenic |  |
| 8 | 170,550,068 | T/C | 0.31 | 8 | Intergenic |  |
| 8 | 173,107,104 | C/A | -1.04 | 8 | GRMZM2G124365 | *Zea mays* B transcriptional activator (b1) |
| 9 | 16,154,952 | G/C | -1.15 | 6 | Intergenic |  |
| 9 | 16,379,663 | CAG/--- | -0.87 | 13 | Intergenic |  |
| 9 | 16,620,539 | A/G | -0.82 | 14 | Intergenic |  |
| 9 | 18,225,380 | C/T | -0.93 | 6 | GRMZM5G899123 | *Zea mays* rust resistance protein rp3-1 (rp3-1) |
| 9 | 99,711,027 | T/C | -1.17 | 10 | Intergenic |  |
| 9 | 128,943,719 | G/A | 0.85 | 11 | GRMZM2G045178 | uncharacterized |
| 9 | 133,801,705 | C/A | 1.38 | 10 | GRMZM2G134279 | *Zea mays* starch branching enzyme IIb (ae) |
| 9 | 142,738,763 | A/G | -0.53 | 9 | GRMZM2G150598 | *Zea mays* starch synthase I (sS1) |
| 9 | 142,906,275 | C/A | -0.54 | 40 | Intergenic |  |
| 9 | 143,305,297 | C/T | -0.51 | 7 | Intergenic |  |
| 9 | 150,278,166 | C/T | 0.75 | 31 | Intergenic |  |
| 10 | 1,429,820 | C/T | 0.39 | 20 | GRMZM2G048067 | uncharacterized |
| 10 | 1,529,739 | C/A | 0.47 | 20 | GRMZM2G098603 | *Zea mays* B73 pathosis-related protein 2 |
| 10 | 1,716,176 | C/T | 0.53 | 15 | GRMZM2G430780 | *Zea mays* NADPH-dependent reductase (a1) |
| 10 | 1,888,649 | C/T | 0.80 | 14 | GRMZM2G022606 | uncharacterized |
| 10 | 1,918,857 | A/T | 0.51 | 14 | GRMZM2G104638 | uncharacterized |
| 10 | 1,922,550 | G/C | 0.36 | 14 | GRMZM2G104655 | Ribosome inactivating protein 1 (rip1) |
| 10 | 3,691,250 | C/G | 0.53 | 6 | GRMZM2G143769 | *Zea mays* rust resistance protein (Rp1-D) |
| 10 | 46,171,212 | G/A | -0.57 | 7 | Intergenic |  |
| 10 | 67,107,876 | C/T | -0.42 | 11 | Intergenic |  |
| 10 | 108,685,826 | A/G | -0.55 | 23 | Intergenic |  |
| 10 | 137,015,827 | G/T | -0.56 | 16 | GRMZM5G818664 | *Zea mays* latency associated nuclear anti |
| 10 | 138,301,336 | C/G | -0.42 | 12 | GRMZM2G105801 | uncharacterized |
| 10 | 144,243,105 | GT/-- | -0.77 | 6 | Intergenic |  |
| 10 | 144,489,188 | G/T | -0.62 | 15 | Intergenic |  |
